# Heart rate variability in adolescents with type 1 diabetes. A cross-sectional study

**DOI:** 10.1007/s00592-026-02747-9

**Published:** 2026-08-04

**Authors:** Minna L. Koivikko, Katri Helttula, Mikko Tulppo, Tytti Pokka, Mika Kallio, Päivi H. Tossavainen

**Affiliations:** 1 Department of Internal Medicine, Oulu University Hospital and University of Oulu, Oulu, Finland; 2 Oulu University Hospital and the Faculty of Medicine, University of Oulu, Oulu, Finland; 3 Research Unit of Biomedicine and Internal Medicine, University of Oulu, Oulu, Finland; 4 Medical Research Center Oulu, Oulu University Hospital and University of Oulu, Oulu, Finland; 5 Research Service Unit, Oulu University Hospital, Oulu, Finland; 6 Research Unit of Clinical Medicine, University of Oulu, Oulu, Finland; 7 Clinical Neurophysiology and Medical Research Center, Oulu University Hospital, Oulu, Finland; 8 Medical Imaging, Physics and Technology, the Faculty of Medicine, University of Oulu, Oulu, Finland; 9 Department of Pediatrics, Research Unit of Clinical Medicine and Medical Research Center Oulu, Oulu University Hospital and University of Oulu, Oulu, Finland

**Keywords:** Child, Adolescent, Diabetes mellitus, Type 1, Heart rate, Autonomic nervous system, Diabetic angiopathies

## Abstract

**Aims:**

We aimed to compare heart rate variability (HRV) between adolescents with type 1 diabetes and healthy age- and sex-matched control participants. We were also interested in how HRV may change during puberty.

**Research design and methods:**

Eighty-five adolescents with type 1 diabetes (mean diabetes duration 7.0 ± 3.6 years) and 103 healthy age- and sex-matched youths consented to this cross-sectional study. We derived HRV data for time domain and power spectra analyses from Holter recordings (mean duration 17.8 h) and analyzed associated risk factors for HRV deterioration during total recording time and nighttime separately.

**Results:**

Total low frequency (LF) and very low frequency (VLF) domains (ms^2^) were significantly lower in children with type 1 diabetes than in healthy controls (LF, *P* = 0.049 and VLF, *P* = 0.005, respectively), whereas no differences were found in the measurements taken at night. The most significant predictors for untoward changes in power spectra were skinfold sum and lipids. Mean heart rate (beats/min), or any of the time domain variables did not differ significantly between study groups. Compared with those in the lowest HbA1c tertile, adolescents with type 1 diabetes in the highest HbA1c tertile had significantly lower LF and VLF power spectra results.

**Conclusions:**

Adolescents with type 1 diabetes have depressed HRV from low to very low frequencies. Deterioration of autonomic nervous system function in adolescents with type 1 diabetes, measured with HRV power spectra is mainly associated with metabolic factors, although it is not possible to establish causal links between these variables.

**Supplementary Information:**

The online version contains supplementary material available at https://doi.org/10.1007/s00592-026-02747-9.

## Introduction

Cardiac autonomic neuropathy (CAN) is associated with an increased risk of cardiovascular mortality [1]. Well-known risk factors for CAN in people with diabetes include long-term poor glycemic control, elevated blood pressure (BP), other microvascular complications and smoking [2, 3]. According to a recent review [4], prevalence of CAN in pediatric type 1 diabetes patients varies between 4% and 39%.

In addition to cardiovascular autonomic reflex tests, newer test modalities, such as baroreflex sensitivity and power spectral analysis of heart rate variability (HRV), have been increasingly used to assess autonomic nervous system (ANS) function in various physiological and pathological settings [5], including type 1 diabetes [6–8].

To date, several HRV-based studies investigating predictive factors for CAN in children and adolescents with type 1 diabetes have been published. CAN in children and adolescents with type 1 diabetes has been related to greater HbA1c variability [9], higher BMI and central adiposity [10, 11] and other metabolic factors [12, 13] In the SEARCH study [6], prevalence of CAN in adolescents with type 1 diabetes was associated with diabetes onset over 10 years of age, elevated BP, and central adiposity.

In the present study, we analyzed long-term day- and nighttime RR recordings from youths with type 1 diabetes and their healthy counterparts. We compared HRV parameters between these two groups of youths and assessed risk factors for ANS dysfunction among adolescents with type 1 diabetes.

## Methods and participants

Children and adolescents with type 1 diabetes were recruited from the Pediatric Diabetes Outpatient Clinic in Northern Ostrobothnia Hospital District, Oulu, Finland, where all patients diagnosed with type 1 diabetes under 16 years of age within local recruitment area are being followed until transition to adult diabetes clinics at approximately 18 years of age. Inclusion criteria were diabetes duration of two years or more and age over 9 years in girls and 10 years in boys. Healthy adolescents were recruited from schools within the same recruitment area. Inclusion criteria for healthy controls were age over 9 years in girls and 10 years in boys without any chronic diseases. All participants and at least one of their guardians provided informed written consent to participate in this cross-sectional study; detailed study protocol has been published previously [14, 15], when original data collection was done i.e. 1997–2003.

Local ethics committee approved the study, and the study was performed according to Good Clinical Practice. Procedures used in this study adhere to the tenets of the Declaration of Helsinki.

All participants were asked to avoid caffeine-containing drinks, cigarette smoking and any vigorous physical activity during study period. One researcher (P.H.T.) examined the participants at the beginning of the study period. The participants stayed overnight in a ward during the study: these arrangements were for easier organization of the study, to stabilize circumstances during recordings, and practical reasons such as long travel distances.

### Anthropometric measurements

Height (cm) was measured with a stadiometer, and body weight (kg) was measured with an electronic scale. Body mass index was calculated as weight in kilograms divided by the square of height in meters. Waist circumference was measured with a nonelastic tape measure to the nearest 0.1 cm. Skinfolds (subscapular, biceps and triceps) were measured with a Harpender skinfold caliper to the nearest 0.1 cm, and the sum of these three measurements was taken as a variable for body fat with a previously described method [16].

Tanner pubertal staging was performed during physical examination, with Tanner pubertal stage 1 being prepuberty; stages 2, 3 and 4 being puberty; and stage 5 being postpuberty.

### Blood pressure measurements

Systolic and diastolic BP (mmHg) were assessed with a random sphygmomanometer using an appropriately sized cuff: systolic BP was assessed on basis of the occurrence of the first Korotkoff sound, and diastolic BP was assessed on basis of the disappearance of the fifth Korotkoff sound.

### Measurement method of heart rate variability

HRV was assessed with Holter recordings during the study period. RR interval recordings were carried out with a portable device (Polar Electro Co Ltd., Kempele, Finland) with a sampling frequency of 1,000 Hz. RR recording started in the afternoon and was continued overnight. The participants made notes on their time of physical activity, bedtime and wake-up (data not shown).

Following HRV parameters were analyzed: heart rate (HR, beats/min); intervals between R waves (RRi, ms); time domain parameters, i.e., SD of all the RR intervals (SDNN, ms); the root mean square of successive differences between normal RRi (RMSSD, ms); and power spectra parameters, i.e., high-frequency power (HF, ms^2^ 0.15–0.4 Hz), low-frequency power (LF, ms^2^ 0.04–0.15 Hz), very low-frequency power (VLF, ms^2^ 0.0033–0.04 Hz) and LF-to-HF ratio. Total recording time was defined as the entire time from start to the end of RR recording, including nighttime. Nighttime recording means a time scale from midnight (24 pm) to 05 am.

### Statistical analysis

Means and standard deviations (SDs) were calculated for continuous variables with a normal distribution. HRV parameters with skewed distributions were analyzed after linear logarithmic transformation and compared between participants with and without diabetes. Spearman’s correlation coefficient was used to explore correlations between variables.

We calculated HbA1c tertiles for participants with type 1 diabetes and compared HRV parameters between the highest and lowest tertiles to clarify the impact of glycemic control on HRV. Differences in HRV parameters between the highest (3rd) and lowest (1st) HbA1c tertiles were assessed with analysis of variance (ANOVA) and Tukey’s post hoc test. The 95% confidence intervals for differences between variable means are shown.

We used multiple linear regression with forward stepwise method to identify associations of age, pubertal development, and metabolic factors such as glycemic control, skinfold sum, lipid profile and BP with HRV parameters. A *P* value < 0.05 was considered to indicate statistical significance. We used IBM SPSS for Windows version 29 (IBM Corp, Armonk, NY, USA) for statistical analyses.

### Data and resource availability

The datasets analyzed in the current study are available from the corresponding author upon reasonable request.

## Results

The present study included 188 participants—85 (44 F; 41 M) adolescents with type 1 diabetes and 103 (53 F; 50 M) healthy age- and sex-matched control subjects—all white Caucasian people. Mean duration of diabetes was 7.0 ± 3.6 years. Mean age was 13.7 ± 1.9 years for youths with type 1 diabetes and 13.6 ± 2.1 years for the healthy controls. Mean HbA1c among participants with diabetes was 8.5 ± 1.6% (73 ± 17 mmol/mol). All adolescents with type 1 diabetes had multiple insulin injection therapy (long acting and rapid acting insulin), and their mean daily insulin dose was 0.92 (SD 0.19) units per kilogram. There were no hypoglycemic episodes in children with diabetes during RR recordings. Standard SMBG measurements were performed on the day of Holter monitoring. Detailed characteristics of study population are shown in Table 1.

**Table 1:** Characteristics of study participants

	Girls			Boys			All		
	Diabetes (*N* = 44)	Healthy (*N* = 53)	95% CI *P* -value	Diabetes (*N* = 41)	Healthy (*N* = 50)	95% CI *P* -value	Diabetes (*N* = 85)	Healthy (*N* = 103)	95% CI *P* -value
Age (years)	13.7 ± 2.1	13.5 ± 2.3	−0.8 to 1.0 0.769	13.8 ± 1.6	13.7 ± 1.8	−0.6 to 0.8 0.789	13.7 ± 1.9	13.6 ± 2.1	−0.5 to 0.7 0.691
Diabetes duration (years)	7.2 ± 3.6	NA		6.8 ± 3.6	NA		7.0 ± 3.6		
Insulin dose (IU/kg/day)	0.91 ± 0.19	NA		0.92 ± 0.19	NA		0.92 ± 0.19		
HbA1c (%)	8.8 ± 1.7	4.9 ± 0.5	**3.3 to 4.3** **< 0.001**	8.3 ± 1.5	5.0 ± 0.4	**2.8 to 3.7** **< 0.001**	8.5 ± 1.6	5.0 ± 0.5	**3.2 to 3.9** **< 0.001**
HbA1c (mmol/mol)	75 ± 18	30 ± 5	**40 to 50** **< 0.001**	71 ± 15	32 ± 5	**36 to 43** **< 0.001**	73 ± 17	31 ± 5	**39 to 46** **< 0.001**
BMI (kg/m^2^)	20.7 ± 2.4	19.2 ± 2.7	**0.4 to 2.5** **0.007**	19.6 ± 1.8	19.0 ± 2.2	−0.3 to 1.4 0.212	20.1 ± 2.2	19.1 ± 2.4	**0.3 to 1.7** **0.004**
Waist to hip - ratio	0.81 ± 0.06	0.78 ± 0.05	**0.00 to 0.04** **0.048**	0.84 ± 0.05	0.84 ± 0.06	−0.03 to 0.02 0.833	0.82 ± 0.06	0.81 ± 0.06	−0.01 to 0.03 0.270
Skinfold sum (mm)	29.6 ± 9.4	24.7 ± 6.9	**1.6 to 8.3 0.004**	20.5 ± 8.3	18.8 ± 8.9	−2.0 to 5.3 0.361	25.1 ± 10.0	21.8 ± 8.4	**0.7 to 6.0** **0.014**
Systolic BP (mmHg)	114 ± 7	112 ± 9	−0.8 to 5.7 0.130	114 ± 7	114 ± 8	−3.7 to 2.9 0.808	114 ± 7	113 ± 8	−1.2 to 3.8 0.349
Diastolic BP (mmHg)	66 ± 8	59 ± 7	**4.5 to 10.5** **< 0.001**	62 ± 8	58 ± 7	**0.9 to 7.1** **0.011**	64 ± 9	58 ± 7	**3.7 to 8.0** **< 0.001**

Data are mean ± SD. 95% CI, 95% confidence interval for the difference between means (independent samples t-test). *BP* blood pressure. (N) number of participants. Systolic and diastolic BP (mmHg) were assessed with a random sphygmomanometer.

Mean length of RR recordings, including nighttime, was 17.5 h (SD 1.7, range 13.0 to 20.5 h) and 18.0 h (SD 1.2, range 14.4 to 20.5 h) in participants with and without diabetes, respectively.

### Mean heart rate

Mean HR (beats/min) during total recording time or during nighttime did not differ significantly between adolescents with type 1 diabetes and healthy youths.

### Time and frequency domain parameters

We found no significant differences in any of HRV time domain parameters between the participants with and without diabetes during total recording time or nighttime recording. LF and VLF frequency parameters (power spectra) were significantly lower in children with type 1 diabetes than in healthy controls during total recording time, whereas there were no such differences in LF, VLF or HF power spectra during nighttime between patients and controls. We have summarized the results of HRV time and frequency domain analysis for the total recording time and nighttime in Table 2.

**Table 2:** Time and frequency domain parameters of HRV in adolescents with and without diabetes

	Girls				Boys				All		
	Diabetes (*N =* 44)	Healthy (*N =* 53)	95% CI	*P*	Diabetes (*N =* 41)	Healthy (*N =* 50)	95% CI	*P*	Diabetes (*N =* 85)	Healthy (*N =* 103)	*P*
Whole registration period											
HR (beats/min)	77 ± 9	76 ± 8	−2.2 to 4.9	0.444	74 ± 9	72 ± 8	−1 to 6	0.216	76 ± 9	74 ± 8	0.166
RRi	787 ± 105	798 ± 86	−50 to 27	0.566	820 ± 103	845 ± 92	−66 to 16	0.224	803 ± 105	821 ± 92	0.210
RMSSD (ms)	64 ± 34	67 ± 27	−15 to 9	0.651	83 ± 40	78 ± 32	−10 to 20	0.523	73 ± 38	72 ± 30	0.864
SDNN (ms)	163 ± 38	168 ± 34	−20 to 9	0.438	182 ± 35	186 ± 48	−22 to 14	0.677	172 ± 38	177 ± 42	0.414
HF ln (ms^2^)	7.19 ± 0.98	7.30 ± 0.78	−0.47 to 0.24	0.52	7.71 ± 0.86	7.61 ± 0.80	−0.25 to 0.44	0.576	7.44 ± 0.96	7.45 ± 0.80	0.917
LF ln (ms^2^)	7.30 ± 0.65	7.50 ± 0.55	−0.45 to 0.04	0.096	7.64 ± 0.52	7.77 ± 0.49	−0.33 to 0.09	0.250	7.46 ± 0.62	7.63 ± 0.53	**0.049**
VLF ln (ms^2^)	7.75 ± 0.53	8.02 ± 0.41	**−0.46 to −0.08**	**0.005**	8.15 ± 0.41	8.27 ± 0.49	−0.32 to 0.07	0.203	7.94 ± 0.52	8.14 ± 0.46	**0.005**
LF to HF -ratio	1.25 ± 0.54	1.33 ± 0.58	−0.31 to 0.14	0.463	1.11 ± 0.85	1.28 ± 0.56	−0.47 to 0.12	0.245	1.18 ± 0.71	1.31 ± 0.57	0.172
Nighttime registration											
HR (beats/min)	66 ± 8	65 ± 8	−1.8 to 5.0	0.385	63 ± 8	60 ± 8	−0.4 to 6	0.090	65 ± 8	62 ± 8	0.079
RRi	921 ± 115	942 ± 118	−68 to 26	0.375	968 ± 122	1014 ± 130	−98 to 8	0.094	944 ± 120	977 ± 129	0.073
RMSSD (ms)	78 ± 41	82 ± 36	−19 to 12	0.627	103 ± 47	97 ± 44	−13 to 25	0.533	90 ± 46	90 ± 41	0.889
SDNN (ms)	115 ± 42	121 ± 41	−23 to 10	0.442	144 ± 36	140 ± 38	−11 to 20	0.598	129 ± 42	130 ± 40	0.813
HF ln (ms^2^)	7.56 ± 1.07	7.64 ± 0.83	0.47 to 0,30	0.661	8.11 ± 0.93	7.97 ± 0.90	−0.25 to 0.52	0.484	7.82 ± 1.04	7.80 ± 0.89	0.885
LF ln (ms^2^)	7.44 ± 0.83	7.63 ± 0.71	−0.50 to 0.12	0.231	7.87 ± 0.60	7.99 ± 0.62	−0.37 to 0.14	0.369	7.65 ± 0.76	7.8 ± 0.69	0.144
VLF ln (ms^2^)	7.94 ± 0.56	8.13 ± 0.46	−0.40 to 0.01	0.065	8.40 ± 0.47	8.47 ± 0.55	−0.29 to 0.14	0.515	8.16 ± 0.56	8.29 ± 0.51	0.094
LF to HF -ratio	1.01 ± 0.55	1.09 ± 0.50	−0.29 to 0.13	0.478	0.95 ± 0.67	1.14 ± 0.52	−0.43 to 0.06	0.137	0.98 ± 0.61	1.11 ± 0.51	0.112

Data are mean ± SD. 95% CI, 95% confidence intervals for difference between means (independent samples t-test). Bolded data are significant *P* values and parameter estimates.

Mean HR correlated negatively with LF power both in adolescents with type 1 diabetes and healthy youths during total recording period (Spearman´s rho values of −0.68 and − 0.63, respectively) and during night recording period (Spearman´s rho values of −0.63 and − 0.55, respectively) (*P* < 0.001).

**Fig. 1:**
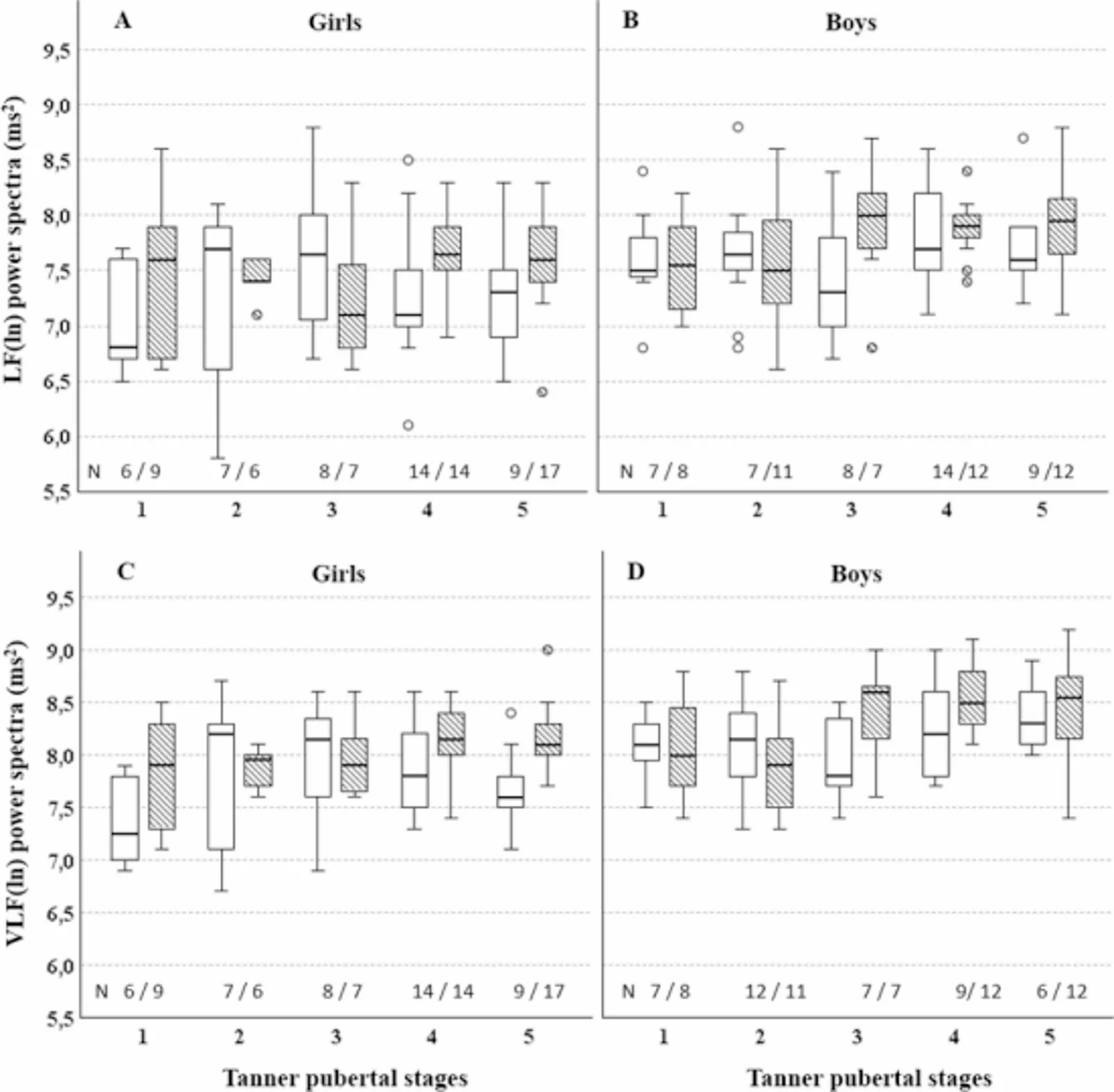
Low frequency (LF) and very low frequency (VLF) power spectra of HRV in adolescents with and without type 1 diabetes. Box and whisker plot; median (line in box), 50% of values (box), lowest and highest value (whiskers). White boxes, type 1 diabetes; striped boxes, healthy adolescents; dots, outliers

Differences in VLF and LF power seemed to deteriorate more in girls than in boys with diabetes compared with their healthy counterparts during pubertal development (Fig. 1). VLF power spectra result for total recording period was significantly lower in girls with type 1 diabetes than in healthy girls (7.66 vs. 8.08, *P* = 0.027, 95% CI −0.80; −0.05) in Tanner stage 5.

### Multiple stepwise regression analysis

Candidate variables in a multiple stepwise regression analysis for predictors of changes in HRV parameters were HbA1c, age, sex, skin fold sum, pubertal stage, daily insulin dose, diabetes duration, systolic and diastolic BP, and lipids. In youths with diabetes negative associations with HRV were observed for skinfold sum, systolic BP, and lipids but not for diabetes duration (Table 3). No such negative associations with metabolic factors were observed in healthy adolescents.

**Table 3:** Associations between HRV parameters and variables in a multiple stepwise regression analysis in participants with and without diabetes

	HR (beats/min)	RMSSD (ms)	SSDN (ms)	HFln	LFln	LF to HF -ratio	VLFln
	β (95% CI) *P*	β (95% CI) *P*	β (95% CI) *P*	β (95% CI) *P*	β (95% CI) *P*	β (95% CI) *P*	β (95% CI) *P*
Adolescents with type 1 diabetes *N* = 82							
HbA1c (/1 unit, %)	**1.2 (0.04; 2.3)** **0.043**	−0.19 0.085	−0.16 0.128	−0.17 0.107	−0.18 0.096	−0.04 0.898	**−0.07 (−0.14; −0.01) 0.023**
HbA1c (/1 unit, mmol/mol)	−0.11 0.715	−0.20 0.074	−0.14 0.189	−0.19 0.074	−0.17 0.115	−0.12 0.860	0.04 0.898
Age (/1 year)	**−1.9 (−2.0; −0.9)** **< 0.001**	−0.1 0.479	0.2 0.099	0.1 0.342	0.1 0.369	0.03 0.762	**0.1 (0.02; 0.12) 0.012**
Skinfold sum (/1 mm)	**0.3 (0.1; 0.5) 0.006**	−0.2 0.083	**−1.0 (−1.8; −0.2) 0.019**	**−0.03 (−0.05;−0.01) 0.007**	**−0.01 (−0.03; 0.0) 0.046**	**0.03 (0.01; 0.04) 0.001**	**−0.02 (−0.03; 0.01)** **< 0.001**
Systolic BP (/1 mmHg)	0.17 0.115	−0.20 0.081	1.00 0.353	**−0.03 (−0.05; 0.00) 0.044**	−0.08 0.485	0.16 0.132	−0.10 0.333
Total chol (/1 mmol/l)	0.1 0.625	**−11.4 (−19.8; −3.0) 0.009**	**−11.4 (−19.5; 2.6) 0.011**	−0.2 0.110	**−0.2 (−0.3; −0.01) 0.031**	0.1 0.685	−0.1 0.362
Triglycerides (/1 mmol/l)	0.07 0.537	−0.1 0.450	−0.22 0.078	−0.18 0.099	−0.04 0.750	0.10 0.333	**−0.2 (−0.3; −0.03) 0.022**
Healthy adolescents *N* = 103							
Age (/1 year)	**−1.2 (−2.0; −0.5) 0.002**	N.S.	N.S.	N.S.	N.S.	N.S.	N.S.
Female vs. male sex	**− 3.5 (−6.4; −0.7) 0.016**	N.S.	**18.0 (2.3; 33.7) 0.025**	N.S.	**0.2 (0.04; 0.4) 0.02**	N.S.	**0.3 (0.1; 0.4)** **0.004**
Tanner stage 1–3 versus 4–5	N.S.	N.S.	**16.9 (0.4; 33.3) 0.045**	N.S.	N.S.	N.S.	**0.2 (0.1; 0.4)** **0.009**
Systolic BP (/1 mmHg)	N.S.	N.S.	N.S.	**0.02 (0.004; 0.04) 0.015**	N.S.	N.S.	N.S.
Total chol (/1 mmol/l)	**2.8 (0.9; 4.9)** **0.007**	N.S.	N.S.	N.S.	**−0.2 (−0.3; 0.02) 0.02**	N.S.	**−0.1 (−0.2; 0.003) 0.044**
HDL chol (/1 mmol/l)	N.S.	N.S.	**−25.1 (−49.9; −0.3) 0.048**	N.S.		N.S.	N.S.

Beta coefficient in multivariate linear regression represents expected change in dependent variable (HR, RMSSD, SSDN, HFln, LFln LF to HF -ratio, VLFln) for one-unit change in a predictor variable (HbA1C, age, skinfold sum, Systolic BP total cholesterol, triglycerides) while holding all other predictors constant. 95% CI, 95%, confidence intervals of β. Skinfold sum was missing from one participant with type 1 diabetes. N.S., non significant (in healthy control group).

### HbA1c tertiles and HRV in participants with diabetes

RMSSD was significantly lower during total recording period, and SSDN was lower at night in the highest (3rd) HbA1c tertile than in the lowest (1st) tertile. LF and VLF power were significantly lower in the highest than in the lowest HbA1c tertile during total recording time (LF, *P* = 0.003 and VLF, *P* = 0.007) and at night (LF, *P* = 0.016 and VLF, *P =* 0.012). HF power tended to decrease with increasing HbA1c during total recording period, whereas LF-to-HF ratio did not change. However, LF-to-HF ratio was lower at night (*P =* 0.045). Data of HRV variables in various HbA1c tertiles data are given in Online Resource 1.

## Discussion

The main findings of the present study were as follows: First, VLF and LF power were significantly lower in adolescents with type 1 diabetes than in their healthy counterparts during long-term RR recording. This phenomenon emerged especially in girls during puberty. Second, decreases in power spectra parameters in participants with diabetes were associated only with metabolic factors such as skinfold sum and lipid levels, whereas in healthy subjects, they were associated mainly with pubertal development, lipid levels and sex. Furthermore, while average HR and time domain parameters did not differ significantly between participants with and without diabetes, in adolescents with poor diabetes control, those parameters and HRV power spectra deteriorated compared with those in participants with diabetes in the lowest HbA1c tertile.

Blunted LF and VLF power spectra in adolescents with type 1 diabetes in the present study were demonstrated during entire recording period, i.e., daytime recording was included, whereas a trend toward deteriorated spectra was found only during nighttime. LF band includes rhythms of periods between 7 and 25 s and is affected by breathing, and reflects merely baroreflex activity during rest [17]. Our finding that HR correlated negatively with LF power both in adolescents with and without diabetes in our study is congruent with previous findings that LF power may reflect more parasympathetic than sympathetic activation in normal or low HR at night.

Adolescent participants adhered to stay overnight in research ward, which verified stable circumstances for measurements during the present study. In our earlier study on conventional cardiovascular reflex tests in young people with diabetes, their VLF power was comparable to that of healthy adolescents in supine position but was blunted during active standing. Similarly, LF power was significantly lower in adolescent participants with diabetes during change from supine to standing [14]. Thus, differences in low-to-very low frequencies of HRV shown in the present study might result mainly from sympathetic modulation of heart without any concomitant vagal withdrawal in participants with diabetes during daytime. Indeed, LF power may paradoxically decrease during high sympathetic activation. This phenomenon has been confirmed in a muscle sympathetic nervous activation (MSNA) experiment published earlier in adults with type 1 diabetes [18], where a negative correlation was observed between sympathetic activation and LF component of HRV. Accordingly, an increase in sympathetic activation without a concomitant vagal withdrawal leads to a decrease in LF power.

The reason why deterioration of power spectra has mostly been demonstrated in adolescent girls with type 1 diabetes is not clear. However, earlier pubertal maturation and more unfavorable metabolic factors in female participants with type 1 diabetes may at least partly explain these differences.

Many of the recent results concerning prevalence of CAN and role of risk factors in type 1 diabetes are derived from large population-based series such as the SEARCH [6, 19] and DCCT/EDIC [20]. There are only a few studies that include long-term HRV analyses or a control group of healthy adolescents for comparison of results [21–23]. In contrast to the results of the present study, Jaiswal et al. [23] reported that SDNN was lower among young participants with type 1 diabetes than among healthy controls during RR recording and that LF power and LF-to-HF-ratio of frequency domain parameters were greater in the context of sympathetic modulation of heart. The fact that the research population with type 1 diabetes in the SEARCH study [23] differs from ours in several aspects, may account for dissenting results and more pronounced changes in the function of ANS in their series. In previous studies, HRV parameters were obtained from a shorter, 4-hour continuous ECG recording, whereas in the present study, a substantially longer recording period was implemented. Another explanation for such differences might be younger age and shorter diabetes duration in our study population. In the study of Jaiswal et al. [23], the mean age of participants with type 1 diabetes was 18.8 years (SD 3.3 years), and duration of diabetes was 9.8 years (SD 3.8 years), whereas in the present study, the mean ages were 13.7 years (SD 1.9 years) and 7.0 years (SD 3.6 years), respectively.

In the most recent studies, predictive factors for CAN in adolescents with diabetes have been investigated. Virk et al. [9] [*n* = 1706, age 15.9 years (range 14.3–17.5), diabetes duration 8.1 years (range 6.3–10.8)] reported that greater HbA1c variability predicts early retinopathy, albuminuria, and CAN after adjusting for known risk factors (HbA1c, diabetes duration, BP and lipids). CAN was assessed by HRV measures obtained from the analysis of 10-minute continuous ECG recordings, and the reference ranges were derived from historical, nondiabetic controls.

Cho et al. [10] examined the predictive role of obesity on CAN in their follow-up study over 7 years, in which 253 participants (with a baseline mean age of 14.4 ± 2.7 years) with a diabetes duration of 7.1 ± 3.7 years and an HbA1c level of 8.3% ± 1.5% (67 ± 16 mmol/mol) were included. They reported that higher BMI and central adiposity were associated with CAN after adjusting for HbA1c. In contrast to those in our study, 33% of subjects in that study were already obese at baseline, which may explain differences in the results between the present study and that of Cho et al. [10].

In our study, although the group mean BMI of adolescent subjects with type 1 diabetes was within the normal range, we found a significant negative association of HRV parameters with skinfold sum, reflecting a possible link with diabetes, obesity and blunted HRV.

In the SEARCH study, Jaiswal et al. [6] reported CAN (≥ 3 abnormal HRV indices) in 12% of 1646 young adults with type 1 diabetes (mean age 19 ± 4 years, diabetes duration 7.9 ± 1.9 years, HbA1c 9.6 ± 2.1%). Emergence of CAN was related to poor metabolic control, as indicated by AUC for HbA1c, high systolic and diastolic BP and elevated triglyceride levels. Compared with our study, in previous study, HRV parameters were obtained from a significantly shorter, 5-minute continuous ECG recording [6]. Similarly, in an earlier study by Jaiswal et al. [23], participants with type 1 diabetes and high glycemic control had significantly lower SDNN and RMSSD values than their peers with better controlled diabetes. There are similarities in findings of our study and these previous studies in relation to high glycemic control in youths with type 1 diabetes and blunted time domain parameters. Optimizing glycemic control and adopting a healthy lifestyle may delay or even prevent progression of CAN in individuals with type 1 diabetes [11, 24]. However, CAN is difficult to assess before development of functional changes in end organs without selective and careful examinations.

HRV can be used to detect CAN in children and adolescents after a relatively short period of type 1 diabetes. Since there is no specific treatment for autonomic neuropathy, the importance of prevention is emphasized by properly treating metabolic risk factors for neuropathy.

Strengths of the present study included a well-described cohort, contemporary controls, and standardized conditions in a hospital ward. Long continuous RR recordings, i.e., 17.5 h and 18 h in participants with and without diabetes, respectively, enabled us to also analyze nighttime HRV.

Limitations include relatively small number of participants, slightly variable length of recordings and cross-sectional study design which cannot provide reliable information on causal relationships between HRV and potential risk factors. Given that the study data were gathered at a time when all the participants were receiving multiple insulin injection therapies and no glucose sensors were available, continuous glucose monitoring and glucose variability data are lacking.

In conclusion, we found changes in cardiac ANS function in adolescents with a relatively short duration of type 1 diabetes. It may be possible to detect CAN in children and adolescents with type 1 diabetes if HRV measurements are used for screening. Since specific treatment options other than sustained good metabolic control are not yet available, early screening and prevention strategies for CAN involving controlling its risk factors are essential.

## Supplementary Information

Below is the link to the electronic supplementary material.

Supplementary Material 1

## Data Availability

Anonymised data are available on reasonable request from the research group.
