# Supplementary material for: Heart rate variability in adolescents with type 1 diabetes. A cross-sectional study
Source: Acta Diabetol. 2026 Aug 4;63(12):2039–47. doi: 10.1007/s00592-026-02747-9 (PMC13769735; doi:10.1007/s00592-026-02747-9)
Supplement: Supplementary file 1 [file 592_2026_2747_MOESM1_ESM.docx]

Acta Diabetologica	20^th^ April 2026

Editorial office

Dear Editor,

Please find enclosed our manuscript “Heart Rate Variability in Adolescents with Type 1 Diabetes. A Cross-Sectional Study” by Minna Koivikko et al.

This report has not been published and is not under consideration for publication elsewhere. The authors do not have any conflicts of interest to disclose.

Sincerely,

Päivi H Tossavainen, MD, PhD

Department of Pediatrics, Research Unit of Clinical Medicine and Medical Research Center Oulu, Oulu University Hospital and University of Oulu, Oulu, Finland

Phone number (personal) +358 503797846; (work) +358 505794446

Email address: [paivi.tossavainen@oulu.fi](mailto:paivi.tossavainen@oulu.fi), [paivi.tossavainen@pohde.fi](mailto:paivi.tossavainen@pohde.fi) and [paivi.tossavainen8@gmail.com](mailto:paivi.tossavainen8@gmail.com)
